# Two melodies in concert: mitral and pulmonary valve replacement late in repaired tetralogy of Fallot

**DOI:** 10.1186/s13019-015-0260-7

**Published:** 2015-04-17

**Authors:** Zhi Fang, Jia Hu, Xianglan Zhu, Ke Lin

**Affiliations:** 1 Department of Cardiovascular Surgery, West China Hospital, Sichuan University, No. 37 Guo Xue Xiang, Chengdu, Sichuan 610041 China; 2 Department of Pathology, West China Hospital, Sichuan University, No. 37 Guo Xue Xiang, Chengdu, Sichuan 610041 China

**Keywords:** Pulmonary regurgitation, Tetralogy of Fallot, Mitral stenosis

## Abstract

Disruption of pulmonary valve integrity after Tetralogy of Fallot repair often results in a cascade of hemodynamic and electrophysiological abnormalities. Here we report an uncommon case of severe pulmonary regurgitation with concomitant rheumatic mitral stenosis diagnosed 25 years after primary Tetralogy of Fallot repair. A 33-year-old man presented with symptomatic palpitation and exercise intolerance and was treated successfully with pulmonary and mitral valve replacement, after which his symptoms improved dramatically.

## Background

Tetralogy of Fallot (TOF) is the most common cyanotic congenital heart defect [1]. The number of adults with a repaired TOF is large. Pulmonary regurgitation (PR) is the major long-term issue in adults after TOF repair [2]. However, few patients have both PR and rheumatic stenosis after repaired TOF, and cases of patients who underwent both pulmonary valve replacement (PVR) for PR after repaired TOF and mitral valve replacement (MVR) are rare. Here we report a case with both PR after repaired TOF and rheumatic mitral stenosis and discuss the choice of prosthesis during PVR and MVR.

## Case presentation

A 33-year-old man was admitted to our hospital with palpitation and lower extremity edema after activity. Six months prior, he started feeling palpitations and more tired than usual after activity. These symptoms became aggravated after he developed a common cold 4 months prior to presentation. He also complained of lower extremity edema and difficulty laying down at night. He was treated with medicines including digoxin and furosemide prior to admission to our ward. The patient underwent TOF repair in our hospital 25 years ago; however, the surgery data were lost and he had no regular follow-up.

**Figure 1:**
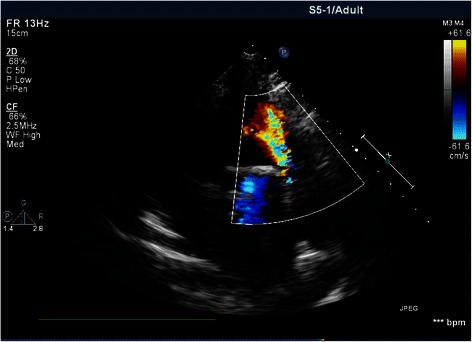
Echocardiogram showing moderate to severe regurgitation within the pulmonary valve.

**Figure 2:**
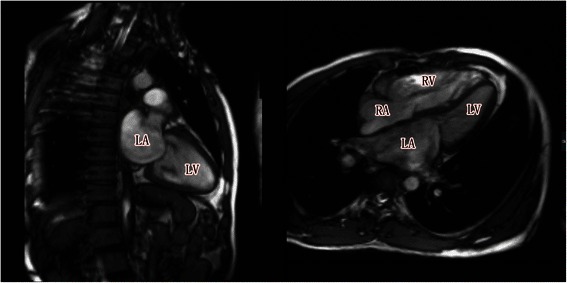
Magnetic resonance image showing thickening and opening restriction of the mitral valve.

On admission, the patient’s heart rate was 67 beats/min, blood pressure was 119/79 mmHg, and respirations were 12 per minute with oxygen saturations around 99% on room air. Under cardiac auscultation, the cardiac rhythm was regular; a diastolic murmur at the apex was heard. Blood chemistry was normal. Echocardiography showed an acceleration of forward blood flow and regurgitation in the pulmonary valve (moderate-severe) (Figure 1), mitral valve stenosis (severe), and tricuspid regurgitation (mild), while a detailed assessment revealed that the pulmonary valve was short and curly. Electrocardiography showed sinus rhythm and a right bundle branch block. Magnetic resonance imaging showed thickening and opening restriction of the mitral valve (Figure 2) as well as mild regurgitation in the tricuspid and aortic valves. Computed tomography showed an increase in texture within the lungs and an enlarged heart.

**Figure 3:**
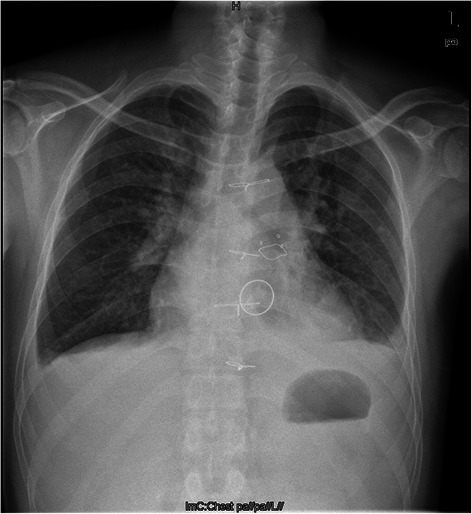
Postoperative X-ray image showing the bioprosthetic valve in the pulmonary valve position and the mechanical valve at the mitral valve position.

The patient was scheduled for mitral valve and PVR surgery after diagnosis. During the procedure, we found that the mitral valve was significantly thickened, suggestive of rheumatic heart disease (RHD). The valve leaflets of the pulmonary valve were significantly shrunken and difficult to repair. The patient underwent a mitral valve replacement with a mechanical valve, and a PVR with a bioprosthetic valve. The patient began to take warfarin from the first postoperative day. The dosage was adjusted according to the International Normalized Ratio (INR) level, which was maintained at about 2.5. A postoperative X-ray showed that the prostheses were well placed (Figure 3). The pathological changes suggestive of RHD were verified by histopathological examination. The patient recovered well and was discharged. On an outpatient visit 6 months after the operation, the patient was graded New York Heart Association functional class I.

### Discussion

TOF is the most common cyanotic congenital heart defect. Patients usually undergo complete repair at 4–6 months of age in most centers [1]. However, problems including PR and recurrent or residual pulmonary stenosis are usually observed in young adults despite surgical repair [1]. Among them, chronic PR is the most common long-term issue in adults after TOF repair [2]. During TOF repair, a transannular incision is usually made to relieve the right ventricular outflow stenosis. Progressive PR may be caused by this repair over a long periods [3]. Severe chronic PR after TOF repair can lead right ventricular dilation, which is associated with right heart failure, exercise intolerance, ventricular arrhythmias, and sudden cardiac death [3,4].

PVR is the most frequent reoperation performed in adults to resolve PR [3,5]. By attenuating the right ventricle volume overload, PVR can lead to improved right ventricular remodeling and function and relieve the clinical symptoms [2,3]. Since a mechanical valve can increase the risk for valve thrombosis, it is not widely used in PVR; rather, the most commonly used valve for PVR is bioprosthetic pulmonary valve [2-5]. However, the life expectancy of the bioprosthetic valve is limited because of issues such as stenosis and calcification. Therefore, we should consider the re-operation rate when choosing the bioprosthetic valve [2-4]. Although the lifespans of different valve types is controversial, Jang et al. reported an acceptable rate of freedom from reoperation for bioprosthetic valves for PVR during the 10-year follow-up period [3].

RHD remains a major health issue in developing countries, where it is the major cause of the cardiovascular mortality and morbidity in young people with a mortality as high as 250000 per year [6]. The main pathological characteristic of RHD is valvular damage, while myocarditis and pericardial are frequently involved [7]. Mitral valve stenosis is the most common valvular lesion in patients with RHD [6]. When the valvular lesions are severe and patients become symptomatic, surgery including mitral valve repair or mitral valve replacement is considered [6]. Mitral valve replacement is warranted when the valvular lesions are too severe to repair. The choice of prosthesis for valve replacement depends on patient characteristics such as the age [6]. According to the guideline from American College of Cardiology/American Heart Association, mechanical valves are more suitable for patients < 65 years old [8]. Mechanical valves are also recommended for young adults with MVR by the European Society of Cardiology and the European Association for Cardio-Thoracic Surgery guideline [9]. In addition, Kaneko et al compared the outcomes of mechanical valve versus bioprosthetic valve in patients < 65 years with MVR. Their results showed longer survival of patients with MVR who received a mechanical valve compared to bioprosthetic valve, which reconfirms the recommendation of mechanical valve use for MVR in patients < 65 years old [10].

Both PR after repaired TOF and rheumatic mitral stenosis are common heart disease, and there are a number of studies about the treatment or valve choice for each disease. However, PR after TOF repair and rheumatic mitral stenosis in the same patient is rare, and few patients have undergone both PVR for PR after TOF repair and mitral valve replacement. Therefore, how to select the appropriate valves remains unknown. In this case, the bioprosthetic valve was chosen for the PVR to decrease the patient’s risk of anti-coagulation and the mechanical valve was chosen for the MVR to decrease the risk of reoperation considering that the patient was 33 years old. However, we are not sure whether our choice in valves could increase the risk of complications caused by anti-coagulation associated with the mechanical valve or the reoperation rate associated with the bioprosthetic valve. The appropriate INR range for this kind of patient is also still uncertain. Further long-time follow-up is needed to clarify these details.

## Conclusions

Patients who undergo both pulmonary valve and mitral valve replacements after TOF repair are rare. While choosing prostheses for such patients, clinicians should consider patient characteristics and balance the risks and benefits of each kind of prosthesis.

## Consent

Written informed consent was obtained from the patient for publication of this case report and any accompanying images. A copy of the written consent is available for review by the Editor-in-Chief of this journal.

## Abbreviations

TOF: Tetralogy of Fallot
PR: Pulmonary regurgitation
PVR: Pulmonary valve replacement
MVR: Mitral valve replacement
RHD: Rheumatic heart disease
